# Sea cucumbers: an emerging system in evo-devo

**DOI:** 10.1186/s13227-023-00220-0

**Published:** 2024-02-17

**Authors:** Margherita Perillo, Rosa Maria Sepe, Periklis Paganos, Alfonso Toscano, Rossella Annunziata

**Affiliations:** 1 Bell Center for Regenerative Biology and Tissue Engineering, Marine Biological Laboratory, 7 MBL St., Woods Hole, MA 02543 USA; 2 Stazione Zoologica Anton Dohrn, Villa Comunale, 80121 Naples, Italy

**Keywords:** Echinoderm, Sea cucumber, Embryo, Larva, Experimental system, Evo-devo

## Abstract

A challenge for evolutionary developmental (evo-devo) biology is to expand the breadth of research organisms used to investigate how animal diversity has evolved through changes in embryonic development. New experimental systems should couple a relevant phylogenetic position with available molecular tools and genomic resources. As a phylum of the sister group to chordates, echinoderms extensively contributed to our knowledge of embryonic patterning, organ development and cell-type evolution. Echinoderms display a variety of larval forms with diverse shapes, making them a suitable group to compare the evolution of embryonic developmental strategies. However, because of the laboratory accessibility and the already available techniques, most studies focus on sea urchins and sea stars mainly. As a comparative approach, the field would benefit from including information on other members of this group, like the sea cucumbers (holothuroids), for which little is known on the molecular basis of their development. Here, we review the spawning and culture methods, the available morphological and molecular information, and the current state of genomic and transcriptomic resources on sea cucumbers. With the goal of making this system accessible to the broader community, we discuss how sea cucumber embryos and larvae can be a powerful system to address the open questions in evo-devo, including understanding the origins of bilaterian structures.

**Supplementary Information:**

The online version contains supplementary material available at 10.1186/s13227-023-00220-0.

## Introduction

**Fig. 1:**
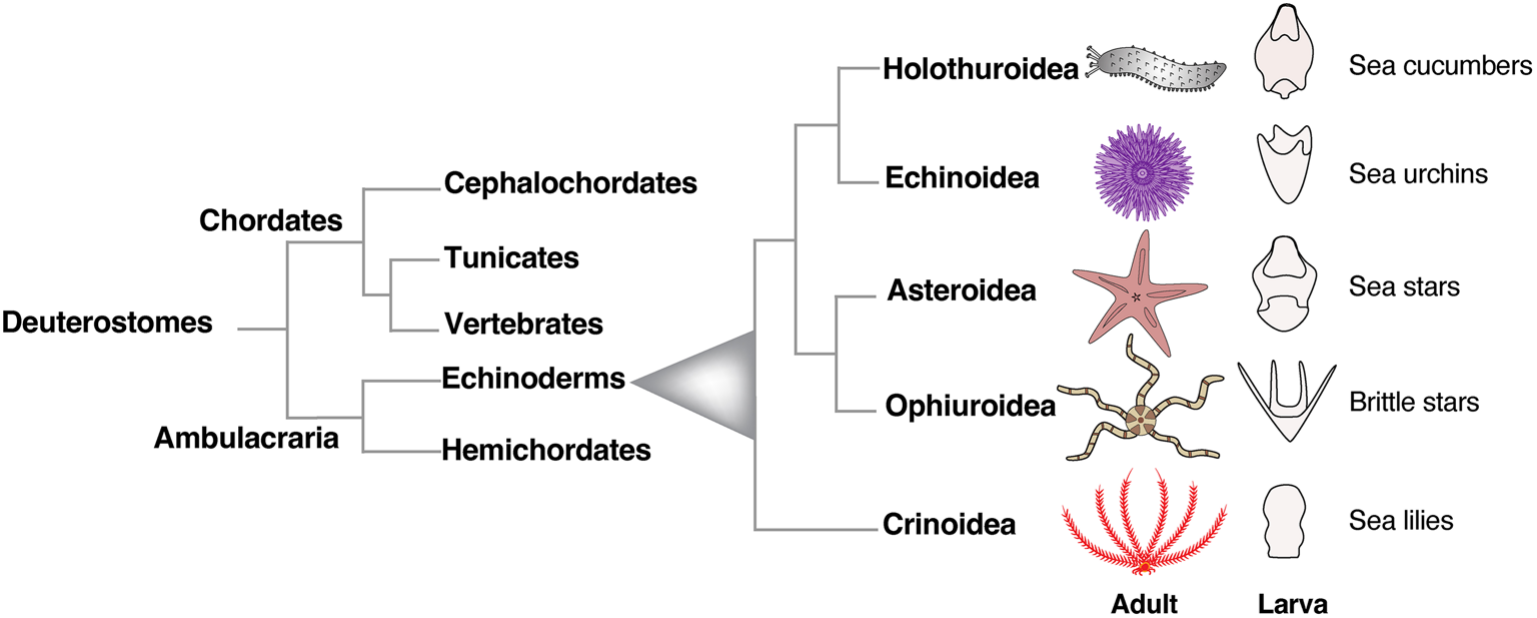
Deuterostomes phylogenetic tree. Phylogenetic relationship of deuterostomes with a focus on living echinoderms. Cartoons represent for each class the typical adult and planktonic larva body plans

Experimental biology with echinoderms has driven major discoveries in the past 100 years, significantly contributing to our understanding of cell, developmental and regulatory biology [24, 41, 46, 72] and reviewed in [5, 14, 15, 32, 65, 67]. This group of animals includes sea urchins, sea stars, sea lilies, brittle stars and sea cucumbers and together with hemichordates belong to ambulacraria, the sister group to chordates (Fig. 1). As with most echinoderms, sea cucumbers are benthic as adults but develop through free-swimming planktonic larvae. Due to their abundance and since they mostly feed on sediment, sea cucumbers dramatically influence sea floor dynamics at different depths, from the intertidal zone to the deep sea [105, 122], and therefore have a high ecological impact. Some sea cucumber species are also considered luxury food in Asia and commercial interest is expanding to species from the Northeast Atlantic and the Mediterranean areas. The consequence of sea cucumbers' increased economic value has led to their illegal and unsustainable fishing to fulfill the market [20, 59], but also prompted many detailed studies for their rearing in animal farms. In fact, although most studies are performed on the adults (including ecotoxicological assessments, isolation of bioactive compounds from adult tissues, exploration of their regeneration capacities, reviewed in [23, 108, 144, 145], there is significant growing interest in dissecting the factors that regulate and/or influence sea cucumbers’ embryonic and larval development, for both aquaculture and basic research purposes.

With the aim of stimulating research on the developmental biology of sea cucumbers, we describe here how different species have been used to study embryology and highlight their potential to boost discoveries in the evo-devo field. First, we provide a compilation of the main spawning methods established in the laboratory for different species and describe their life cycles depicting the main embryonic and larval anatomical features. We then focus on cell type specification looking at all available gene expression studies in embryos and larvae. Finally, we describe all the publicly available genomic and transcriptomic resources and the established experimental techniques to explore sea cucumber cellular and developmental biology.

## Adult body anatomy and spawning methods

Echinoderms display a variety of body shapes, from the spherical sea urchins to the central disk with arms in sea stars and brittle stars, or the central stalk with arms found in the sea lilies (Fig. 1). While other animals in this family display a more overtly pentaradial symmetry, the body of adult holothurians (commonly called sea cucumbers) shows bilateral features, resembling an elongated cylinder with the mouth and the anus located at the opposite ends. At a first look, external pentamery seems limited to the arrangements of buccal tentacles; however, the organization of the internal organs (radial canals, radial nerve cords, muscles) follows a pentameral symmetry, with the exception of the gonad, the madreporite and the digestive tract [133].

Here we describe the most common features of the adult sea cucumbers, but a more detailed description of unique characteristics for each species can be found elsewhere [81, 95, 134, 140]. Adults are usually 10–30 cm long and the body wall can be dark or multicolored, smooth or covered in spines or warty protuberances. Animals in the genera Holothuria, Curcumaria and Stichopus move on the substrate with ventral podia like sea stars, others like Apodida and Molpadida, completely lack podia and are buried in the sediment. Some representatives of the deep-sea Elasipodida use their enlarged podia to walk and some species are able to swim [37, 95, 133, 141]. The mouth is located at one of the two extremities and it is surrounded by tentacles that are part of the water-vascular system, an organ composed of a series of canals with locomotor functions. The mouth opens into a muscular pharynx followed by the digestive system that is mainly formed by a long, looped intestine [74]. If threatened, sea cucumbers can expel their entire gut and enteric nervous system, which are then regenerated after a few weeks, making these animals an excellent model to study intestine and nervous system regeneration [38, 115, 123, 124]. In addition, while the majority reproduce sexually, some holothurian species are capable of asexual reproduction through fission [45, 151].

Another unique feature of sea cucumbers among the echinoderms is that they possess a single gonad and a single gonopore instead of five, located anteriorly at the base of the tentacles [133]. Like other echinoderms, gametes are released in the sea water [133]. Compared to the broad information on the reproductive biology of echinoids and asteroids, less is known in holothurians likely due to the difficulties of artificially spawning the adults. However, researchers found ways to spawn selected species by stressing the animals and triggering their gonads to mature their oocytes and spawn. The major spawning techniques involve mechanical (dry and light stress), thermal and chemical treatments, and are summarized below.

*Apostichopus japonicus*, *Stichopus horrens* and *Holothuria scabra* can be induced to spawn by dry stimulation, meaning the animals are first kept out of seawater for 30 min and subsequently exposed to a jet of sea water [1, 73, 131]. Another spawning method combines light stress and temperature shock, when animals are left in the dark at temperatures that are ~ 5 °C higher than natural seawater [76]. *Holothuria polii* and *Holothuria scabra* can be induced to spawn with thermal shock by raising the sea water temperature by 3–5 degrees for 1–2 h, followed by placing the animals at the optimal temperature [106, 127]. In contrast, a combination of thermal shock and thermal stimulation (consisting of gradually increasing the water temperature by 3 °C and after a day applying a thermal shock by quickly further raising the temperature by 3 °C and returning it back to the previous conditions) is effective in the Mediterranean *Holothuria tubulosa* [126]. For some species, oocyte maturation can be achieved by chemical treatments, one example being gonad stimulation with radial nerve extracts [79]. Oocyte maturation and spawning in *H. scabra* and *Holothuria leucospilota* is induced by injecting into the body wall (or by bathing their gonads inside) sea water with a recombinant relaxin-like gonad-stimulating peptide [36]. Another example is the protein thioredoxin that has been successfully used for oocyte maturation in *H. tubulosa* and *H. scabra* [86]. Finally, *A. japonicus* oocyte maturation is induced by bathing the gonad in sea water containing the neuropeptide cubifrin [164]. Besides these few examples, a hormone capable of inducing spawning for all the species is lacking. Therefore, when approaching a new species, several methods need to be tested.

## Embryonic development and metamorphosis

**Fig. 2:**
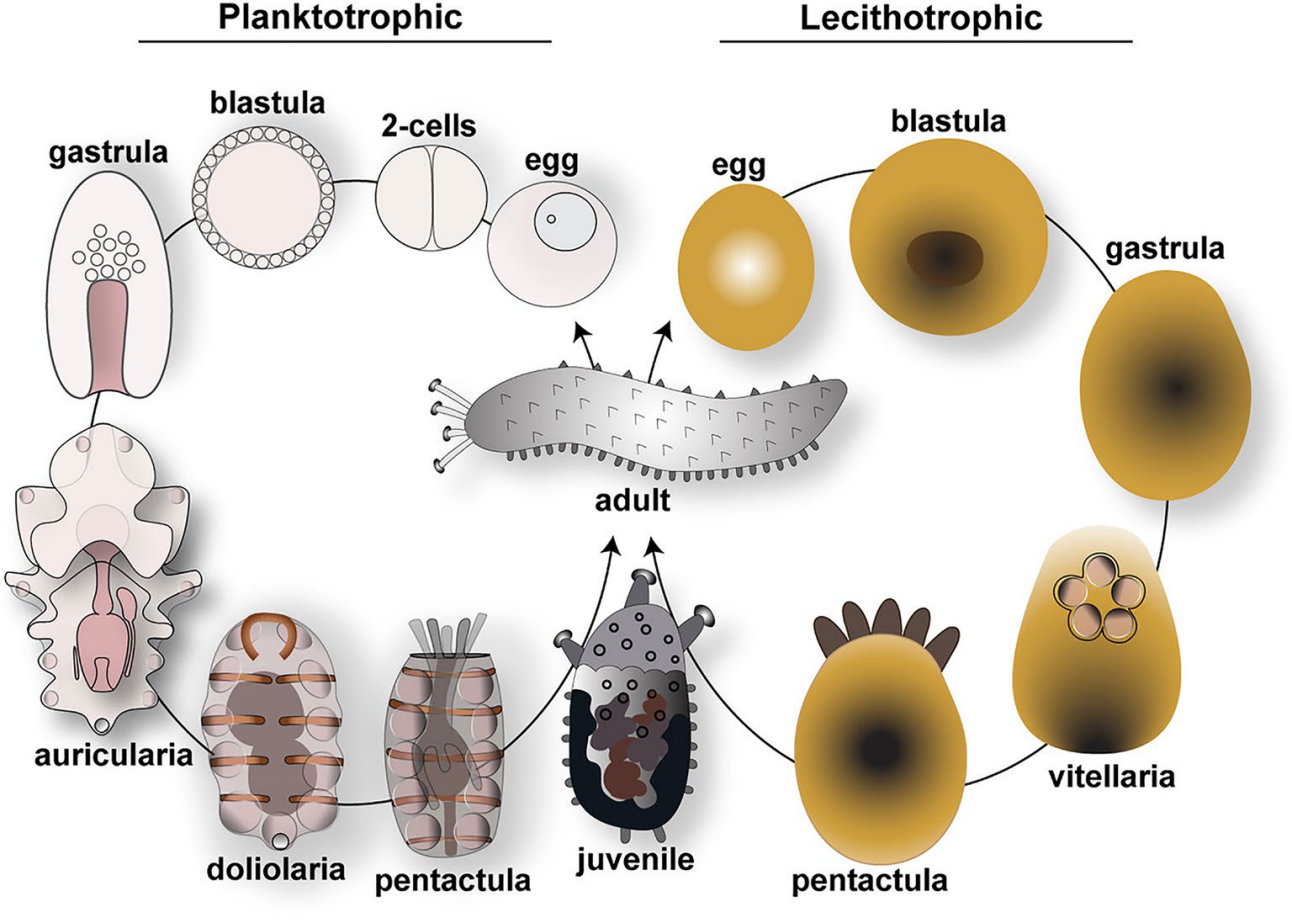
Life cycle of sea cucumbers. Schematic showing the two types of reproductive strategies of Holothurians. Planktotrophic species have transparent embryos and develop through a feeding larva stage, while lecithotrophic species have yolky embryos that do not develop a complete digestive system and do not feed

As with most benthic marine invertebrates, embryos of different species of holothurians can either develop through a dispersive planktonic larval stage or be brooded in adult bodies [116]. Free-living larvae can be planktotrophic or lecithotrophic (Fig. 2). Planktotrophic larvae are optically transparent, develop a fully functional digestive system to feed in the plankton, and progress through the stages of auricularia, doliolaria and pentactula. Lecithotrophic larvae have opaque non-feeding embryos that rely on maternal yolk reserve to grow through the larval stages of vitellaria and pentactula. Through attempts at setting up new systems for aquaculture, the embryonic development of several holothurian species has been described in detail. Here, we summarize the main developmental stages of species with planktotrophic larvae, such as *Holothuria forskali*, *A. japonicus*, *S. horrens*, *H. tubulosa* and of species with lecithotrophic larvae like *Athyonidium chiliensis* and *Curcumaria frondosa.* For detailed information, Table 1 reports the geographic area, the temperature conditions and the developmental timing for the most studied species together with literature references.

**Table 1:** Embryonic and larval development of the main species of sea cucumbers used for evo-devo studies or aquaculture

Species	Area	*T* (°C)	2 cells (min)	4 cells (min)	Blastula (h)	Gastrula (h)	Auricularia (early–late, days)	Doliolaria (days)	Pentactula (days)	Juvenile (days)	Refs.
Planktotrophic											
*Holothuria atra*	Indo-Pacific	30	60–120	180–240	24	na	2–10	20	na	na	[128]
*Holothuria tubulosa*	Atlantic NE, Mediterranean	24	110	150	12	20	3–20	24	26	27–30	[126]
*Holothuria polii*	Mediterranean	24	90	120	12	20	3–7	8–9	9–10	15–90	[127]
*Holothuria forskali*	Atlantic NE, Mediterranean	17	120	240	24	36	4–35	36	42	43–120	[85]
*Holothuria scabra*	Indo-Pacific	25–27	60	120	5	12–14	2–12	12–13	13–15	14–17	[129]
*Apostichopus japonicus*	Pacific NW (Japan, Korea, China)	20–23	60	120	7–12	24	2–11	11–13	12–17	11–23	[166]
*Stichopus horrens*	Indo-Pacific	25–27	40–50	70–80	3,6	25	2–13	18–26	19–27	30	[73]
*Parastichopus californicus*	Pacific NE	10–12	240	360	24	40	6–13	60	24-48 h post doliolaria	50d after settlement	[29]
*Australostichopus mollis*	New Zealand, South Australia	18	40–60	na	5–6	25–36	56–16	18–20	21–23	24–27	[142]
Lecithotrophic											
*Athyonidium chilensis*	Pacific SE (Chile)	13	60–180	180–300	24–25	48–49	–	4–5	7	21–35	[60]
*Curcumaria frondosa*	North Atlantic (Canada, Maine)	0–13	390±60	510±78	48±3.6	72±8.5	–	8±1	11±1.5	46±2	[62]

### Developmental stages of planktotrophic species

**Fig. 3:**
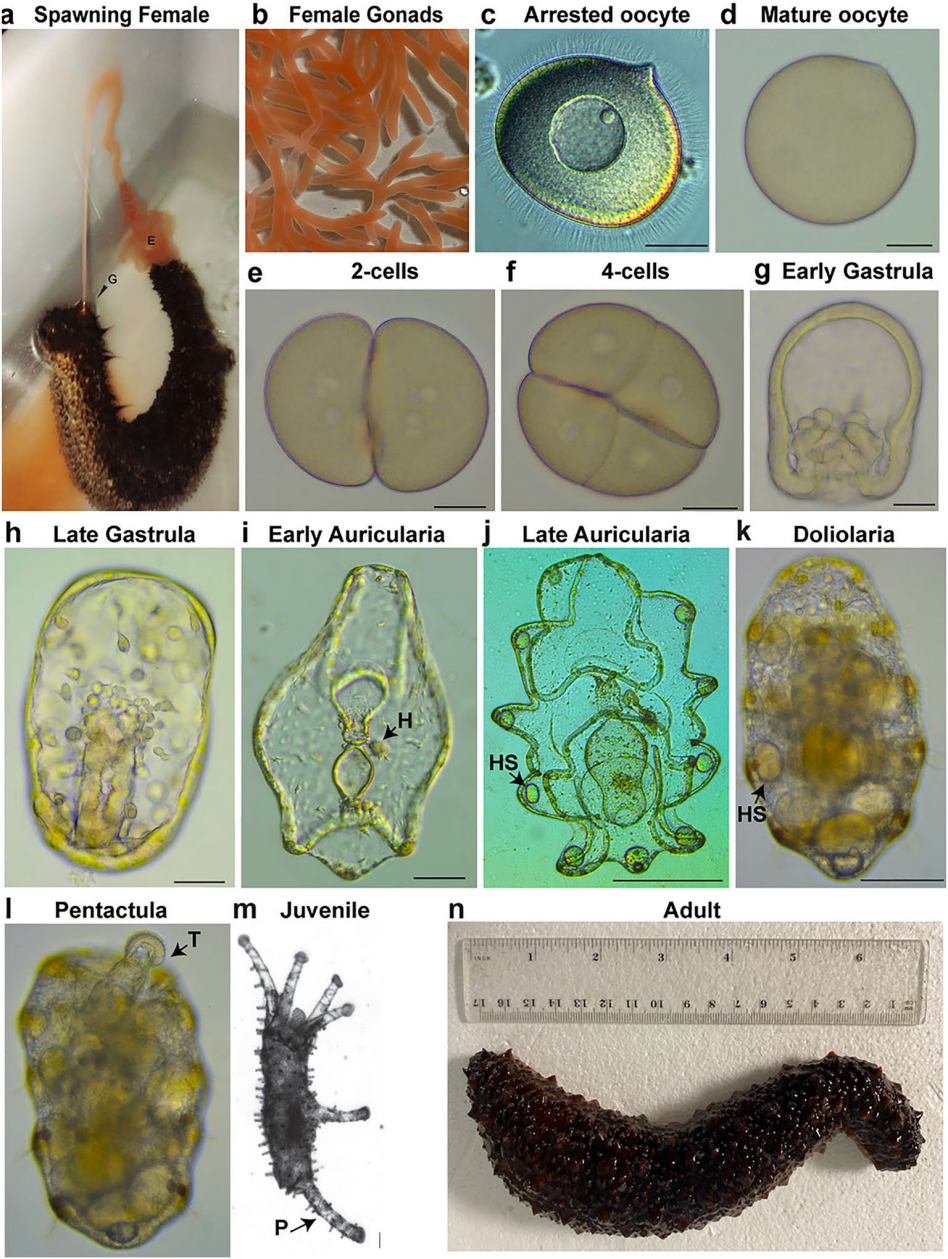
Development and morphology of Holothuroidea, focus on *H. tubulosa*. **a** Spawning female (G, gonopore; E, eggs); **b** female gonads; **c** arrested oocyte, note the nucleus in the center; **d** mature oocyte, note that the nucleus is not visible anymore; **e** 2-cell stage; **f** 4-cell stage; **g** early gastrula and **h** late gastrula; **i** early and **j** late auricularia larvae; **k** fully developed doliolaria; **l** early pentactula larva. **m** Juvenile and **n** adult (**b**–**i** scale bar = 40 μm; **j**–**m** scale bar = 100 μm). H, hydropore; HS, hyaline spheres; T, tentacles; P, podia. **a**, **m** are modified from [126], **b**, **c**, **d**, **e**, **f**, **g**, **h**, **i**, **j**, **k**, **I**, **n** are original pictures taken in the Annunziata’s laboratory

**Fig. 4:**
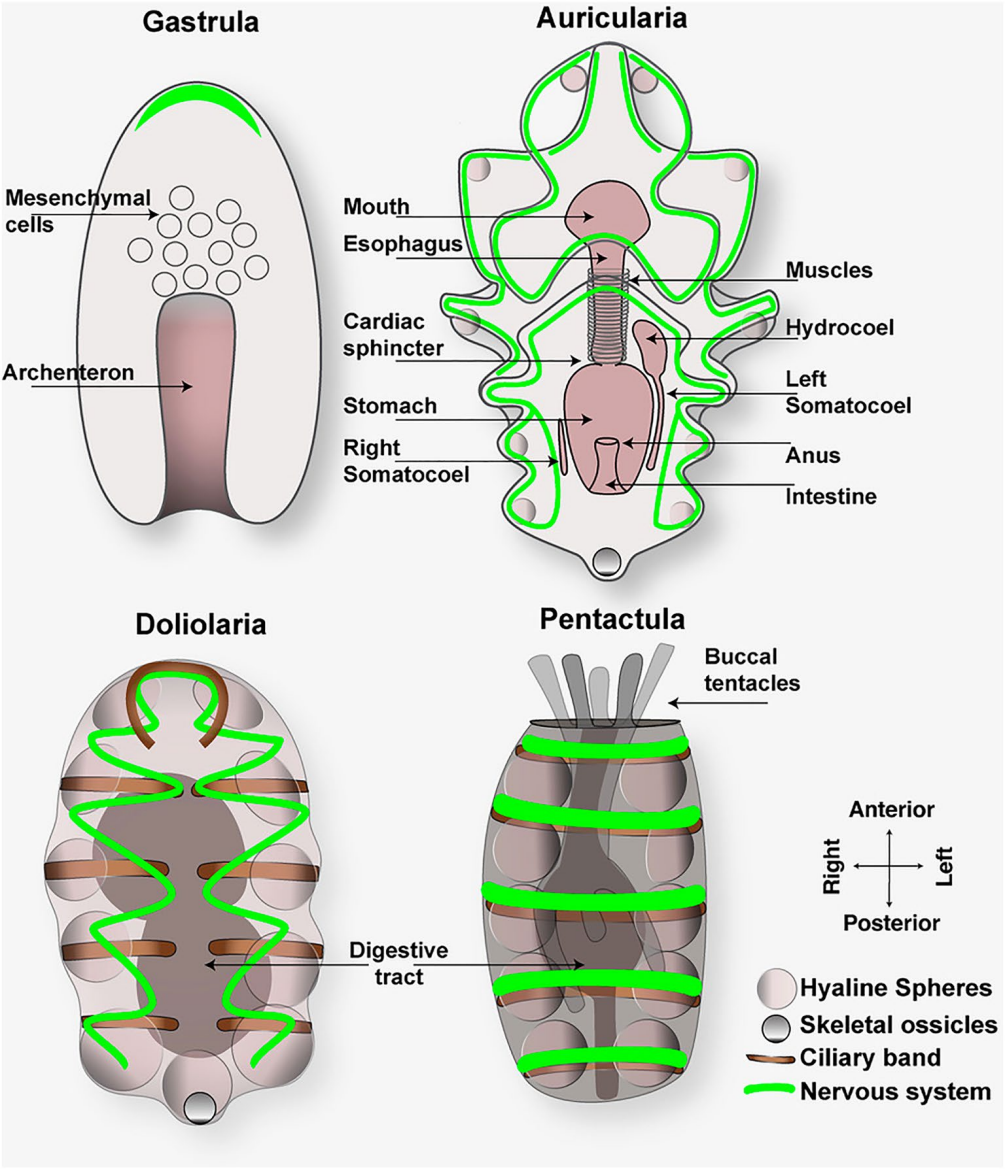
Cartoons showing the morphology of gastrula, auricularia, doliolaria and pentactula larvae. In the gastrula, mesenchymal cells migrate from the archenteron (future gut) while it elongates anteriorly. The first neurons appear at this stage. Auricularia larvae are characterized by a functional digestive system, the presence of the hydrocoel, the left and right somatocoels and hyaline spheres. At this stage, the nervous system increases in complexity. Doliolaria is a transitional barrel-shaped larva that does not feed. It has larger hyaline spheres compared to the auricularia. Adult organs are formed in the pentactula, the last stage before the juvenile. In figure, a green continuous line indicates the neurons underneath the ciliary band. To make the auricularia cartoon clearer and show distribution of neurons we omitted the ciliary band that follows the same pattern

In sea cucumbers fertilization and embryonic development occur externally. Eggs are often spherical and transparent for planktotrophic species and are generally large, opaque and oval for lecithotrophic ones (Fig. 2). Spawning behaviors of holothurians are unique and help researchers identify males from females: when ready, males spawn first by moving close to the water surface and adopting a standing position, followed an hour later by females who lift their anterior side and release eggs [126], Fig. 3a). Ovaries are a branched, tubular structure, usually of a transparent pink or orange color (Fig. 3b). Prophase 1 arrested oocytes have a clear nucleus (Fig. 3c) that breaks down when the oocyte is mature and ready to be fertilized (Fig. 3d). After fertilization, uniform radial cleavages are observed (Fig. 3e and f), followed by the blastula stage, a spherical embryo with a large blastocoel and the surface covered with cilia [126]. After hatching from the fertilization membrane, blastulae are the first swimming stage that subsequently elongate along the anterior–posterior axis to form the gastrula (Fig. 3g). Gastrulation proceeds through invagination of the archenteron, followed by migration of mesenchymal cells from the tip of the archenteron, the future gut (Fig. 3h). By late gastrula stage, the mouth opens, and the site of archenteron invagination becomes the anus. The first neurons start to appear, some of which have been identified as serotonergic neurons located in the anterior ectoderm [110]. The gastrulae elongate to form the auricularia larva (named by Johannes Muller in 1840 based on the resemblance with human ears), the first planktotrophic larval stage. Auriculariae are transparent and bilaterally symmetric larvae (Fig. 3i). The fully formed digestive tract is divided into three regions: a muscular esophagus, a stomach that is separated from the esophagus by the cardiac sphincter, and the intestine that leads to the anus (cartoon in Fig. 4). While swimming in the ocean, auricularia larvae grow in size and sense the external environment thanks to an extensive nervous system that interconnects with the looped ciliary bands (a portion of the ectoderm made of cells with a single cilium) to control feeding and locomotion [143]. Underneath the ciliary bands is a continuous strip of nerve fibers characterized by serotonergic neurons lined along the ventral and dorsal anterior ectoderm of the larva [27, 110] (Fig. 4). On the left of the digestive system, tubular tissues composed of the hydrocoel (Fig. 3i, arrow) and the left somatocoel appear; a smaller somatocoel is also formed on the right side [17] (Fig. 4). Auricularia from different species can be distinguished by the folding of the posterior ectoderm: the larvae belonging to the genus *Holothuria* have a triangular protrusion, while the *Stichopus* larvae have a flat posterior end [78, 169]. Near the posterior end appears the primordium of a larval skeleton made of simple ossicles (number varies based on the genus [134]) that are probably used to keep the larva balanced in the water column [117]. At this stage, the hyaline spheres appear in the larval arms [34] (Fig. 3j, arrow). Hyaline spheres are refractile structures unique to holothurians, which increase in size during feeding and represent a nutrient storage of neutral lipids that larvae use for metamorphosis, since during this transformation phase they are not able to feed [120].

Developmental differences of holothuroids with other classes of echinoderms are evident also at metamorphosis. While in the other echinoderms metamorphosis starts with the formation of the juvenile rudiment on the left side of the stomach, in holothurians there is no rudiment and metamorphosis consists in the reorganization of the larval structures into the juvenile body plan [125, 169]. During metamorphosis larvae go through a transitional barrel-shaped doliolaria stage that is smaller in size than the auricularia and does not feed (Fig. 3k). In the doliolaria larva, the ciliary band breaks to form five transverse bands, the hyaline spheres increase in size (Fig. 3k, arrow), and the digestive tract is rearranged [138]. When the primary tentacles appear, larvae swim close to the substratum and drop to the bottom [126, 127]. At this point, the doliolaria transforms into pentactula, a benthic translucent larva that uses its five tentacles to attach to the substratum and, in some species, podia for locomotion (Fig. 3l) [51, 78]. The definitive adult organs form at the pentactula stage, the last larval stage before growing into the newly settled juvenile (Fig. 3m) that resembles the adult body plan (Fig. 3n).

### Lecithotrophic species development

Lecithotrophic larvae differ from planktotrophic larvae as they skip the auricularia and doliolaria stages and do not require food to complete metamorphosis and become pentactulae (Fig. 2). Species in this group include for instance *C. frondosa*, *A. chilensis and Psolus holothuroids* [60, 62, 91, 102]. Eggs are large and yolky, embryos are opaque and internal structures are not visible. Bean-shaped gastrulae develop into barrel-shaped non-feeding vitellaria larvae [139] that lack hyaline spheres and some species do not form skeletal rods until the pentactula stage [78]. The most visible structure in the vitellaria larva is the vestibule, an opening on the anterior end from where the five primary buccal tentacles will eventually protrude in the pentactula stage [57, 60, 62] (Fig. 2). Similar to the planktotrophic species, the lecithotrophic ones develop into a juvenile sea cucumber that resembles the adult animal (Fig. 2).

Some lecithotrophic holothurians reproduce by brooding of the embryos on the body of the adults, a reproduction strategy that has been observed in numerous species [74, 139]. Examples are the cucumariid holothuroid *Neocnus incubans* [4], the deep-sea Holothurian *Oneirophanta mutabilis* [63], and the Atlantic Ocean *Psolus patagonicus* [58]. The great variety of reproductive strategies in holothurians is highlighted by the recent discovery that brooding in *Holothuria floridana* is facultative since the exceptionally sticky embryos can benefit from growing on the adult body wall but can also live in the plankton [132].

## Cell type diversity and evolution in holothurians

Being the sister group of chordates, echinoderms have been extensively studied to understand the cellular and molecular regulation of development, allowing evolutionary comparisons with other deuterostomes, including vertebrates. The rich morphological diversity of their larvae makes echinoderms important research organisms to address how evolutionary novelties arise [14]. Indeed, the feeding larvae of echinoderms can be distinguished morphologically into two main types, the pluteus-like larvae of echinoids and ophiuroids, and the auricularia-like larvae of holothuroids and asteroids. Sea urchins and sea stars are often used in evo-devo studies addressing the evolution of organs such as the mineralized skeleton, the digestive tract and the nervous system of the larvae. Because of their larval morphology that shares traits with both sea urchins (e.g., the presence of a skeletogenic cell type [101] and sea star larvae (e.g., the overall external shape of the auricularia stage), the sea cucumber is emerging as a valuable experimental system to assess cell type evolution. In addition, the holothurian developmental strategy of developing into a feeding auricularia followed by a doliolaria is considered as ancestral for echinoderms [121, 125]. In this section, we provide an overview of the evo-devo discoveries in this emerging experimental system (in particular in *A. japonicus, Parastichopus parvimensis, Parastichopus californicus, H. scabra* and *Holothuria atra*) and the future potential of studies in holothurians to unravel the evolution of novel structures.

### Antero-posterior patterning

A distinctive trait of echinoderms is the shift in their life cycle from a larva with bilateral symmetry to an adult with a pentameric body plan. However, while the adults of most echinoderms do not show a clear antero-posterior (AP) axis, sea cucumbers have an elongated body with the oral (anterior) and the anal (posterior) regions at opposite ends and the only overt sign of external pentamery are the five buccal tentacles. On the other side, the internal organs (radial canals, radial nerve cords, muscles) of adult sea cucumbers are arranged following a clear fivefold distribution with the exception of the gonad, the madreporite and the digestive tract.

Because of the torsion of the coelomic sacs during metamorphosis, the oral/aboral (OA) axis of the juvenile and adult does not correspond to the OA axis of the larvae in echinoids, asteroids and crinoids [121]. This torsion does not occur in holothuroids [74, 153] and the adult OA axis thus seems to correspond to the OA axis of the larva [138], although this is still a contentious issue. The absence of the torsion of the larval axis in holothuroids makes easier to follow the axis generation in the cylindrical and seemingly bilateral body plan of the adults. Thus, while it is critical to study all the five groups of echinoderms—including fossil records—to understand stem echinoderm features, holothuroids represent a valid group to study the molecular regulation and the evolution of adult axis emergence.

*Hox* genes are known for their crucial role in body patterning. One main unique feature of *hox* genes is that they are clustered in the genome and their spatial sequence of activation along the AP axis of the body follows their relative position along chromosomes, a property named spatial collinearity [71]. Despite the remarkable number of studies exploring *hox* genes, several aspects of their regulatory mechanisms and the evolution of their functions are still unclear [48, 94]. Because of the shift from bilateral to pentameric symmetry during their life cycles, echinoderms are exceptional systems to study *hox* gene evolution [28, 40, 53]. The analysis of *hox* genomic organization in *Strongylocentrotus purpuratus* [30] and more recently in *Paracentrotus lividus* [97] has revealed a reorganization of the *hox* cluster in echinoids, with translocation and inversion of the anterior *hox* class genes. These rearrangements were proposed as responsible for the bilateral symmetry break in echinoderms [40]. However, the finding of an intact *hox* gene cluster in the genome of the sea star *Acanthaster planci* [18] and more recently in the genome of the sea cucumber *A. japonicus* [173] disproved this hypothesis. On the other side, data on *hox* gene expression in sea urchins [7, 13], sea lilies [64] and sand dollars [152] showed that *hox* genes are sequentially expressed along the AP axis of the late larval somatocoels. Finally, a recent work on the sea star *Patiria miniata* highlights the sequential expression of *hox* genes in the mesoderm and endoderm of the adult body [53]. Together, these data show that *hox* genes have a role in the AP patterning of structures present in the echinoderm late larvae and adults while they are not involved, as a group, in the AP patterning of the embryonic stages [13].

**Fig. 5:**
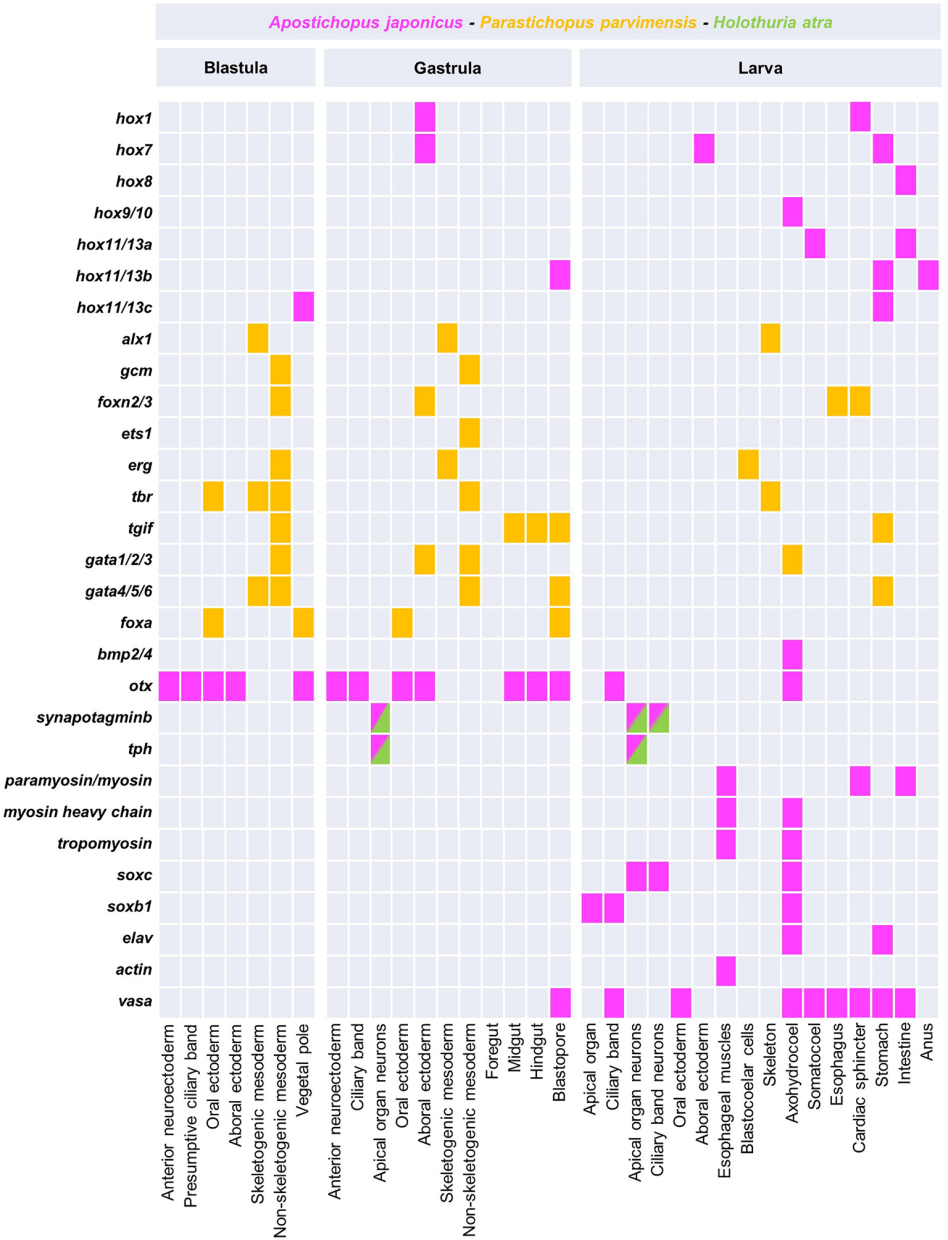
Square plot summarizing all the gene expression data available for sea cucumber embryos and larvae. Colors represent the species where gene expression was investigated. Tph and Synaptotagmin expression has been inferred based on the localization of the proteins through immunohistochemistry

A different scenario has been found in the sea cucumber *A. japonicus* with *hox* genes expressed sequentially also at embryonic stages. In particular, five *hox* genes (*hox1*, *hox7*, *hox8*, *hox11/13a*, and *hox11/13b*) are expressed in sequence along the gut of early larval stages and eight *hox* genes (*hox1*, *hox5*, *hox7*, *hox8*, *hox9/10*, *hox11/13a*, *hox11/13b*, and *hox11/13c*) show a similar expression in doliolaria and pentactula stages (Fig. 5) [82]. This represents the first example of *hox* genes expressed as a group in early developmental stages in echinoderms, following a pattern similar to that found in other bilaterians and makes sea cucumbers a suitable experimental system to study the role of Hox genes cluster in the formation of embryonic and adult structures in echinoderms.

Another group of genes involved in AP patterning of embryonic structures are the *parahox* genes. Like *hox* genes, *parahox* are clustered in the genome and function following spatial collinearity (genes at the 3ʹ of the cluster are expressed anteriorly, genes at the 5ʹ are expressed posteriorly) and temporal collinearity (anterior genes are expressed earlier and posterior genes are expressed later) [26]. While sea urchins seem to have lost this type of genomic organization [16], an intact *parahox* cluster has been found in asteroids [11, 18] and in the sea cucumber *A. japonicus* [173]. In addition, temporal collinearity has been described for *parahox* gene expression during the development of both the sea star and the sea cucumber [11, 173]. It is intriguing that two of the *parahox* genes (*pdx* and *cdx*) are expressed in a similar way showing nested domains along the AP axis of the sea urchin and sea star larval guts, despite the different genomic organization in the sea urchin [16]. It will be interesting to explore the expression and function of *parahox* genes in the sea cucumber larva, to address the role of cluster organization in the regulation of their expression and the conservation/divergence of their function in gut patterning in echinoderms [9, 10, 12].

### Nervous system organization in holothurian larvae

The nervous system of echinoderm larvae consists of two main structures: the apical organ, that is hypothesized to act as the central nervous system of the larva, and the ciliary band neurons representing the peripheral nervous system [21]. The architecture of the echinoderm nervous system has been the subject of many studies carried out mainly in sea urchins and sea stars through immunohistochemistry, in situ hybridization and RNA-seq. Such studies have revealed the presence of conserved neuronal subtypes, including photoreceptor cells, sensory neurons and neuropeptide producing cells, tracing their evolutionary history to non-chordate deuterostomes. Moreover, perturbation analyses led to the identification of the gene regulatory networks (GRNs) controlling neuronal specification and differentiation, providing insights into the distinct differentiation steps taking place during development.

Studies based on immunohistochemical detection of the echinoderm specific pan-neuronal marker Synaptotagmin B in sea cucumbers showed that the first neurons appear at the late gastrula stage in the anterior neuroectoderm domain [22, 110, 178] (Fig. 4). These neurons arise from the thickened anterior neuroectodermal epithelium (a feature shared with echinoids) and are immunoreactive to serotonin, as also observed in most echinoderms at equivalent developmental stages, supporting the proposed crucial role of serotonin in the apical organ of marine larvae in controlling swimming behavior and locomotion [39, 96]. In addition, *soxB1* and *soxC* orthologues, that are essential for the specification of neuronal precursors in sea urchin and sea stars [8], are expressed in similar domains during sea cucumber development (Fig. 5) [154, 155].

**Fig. 6:**
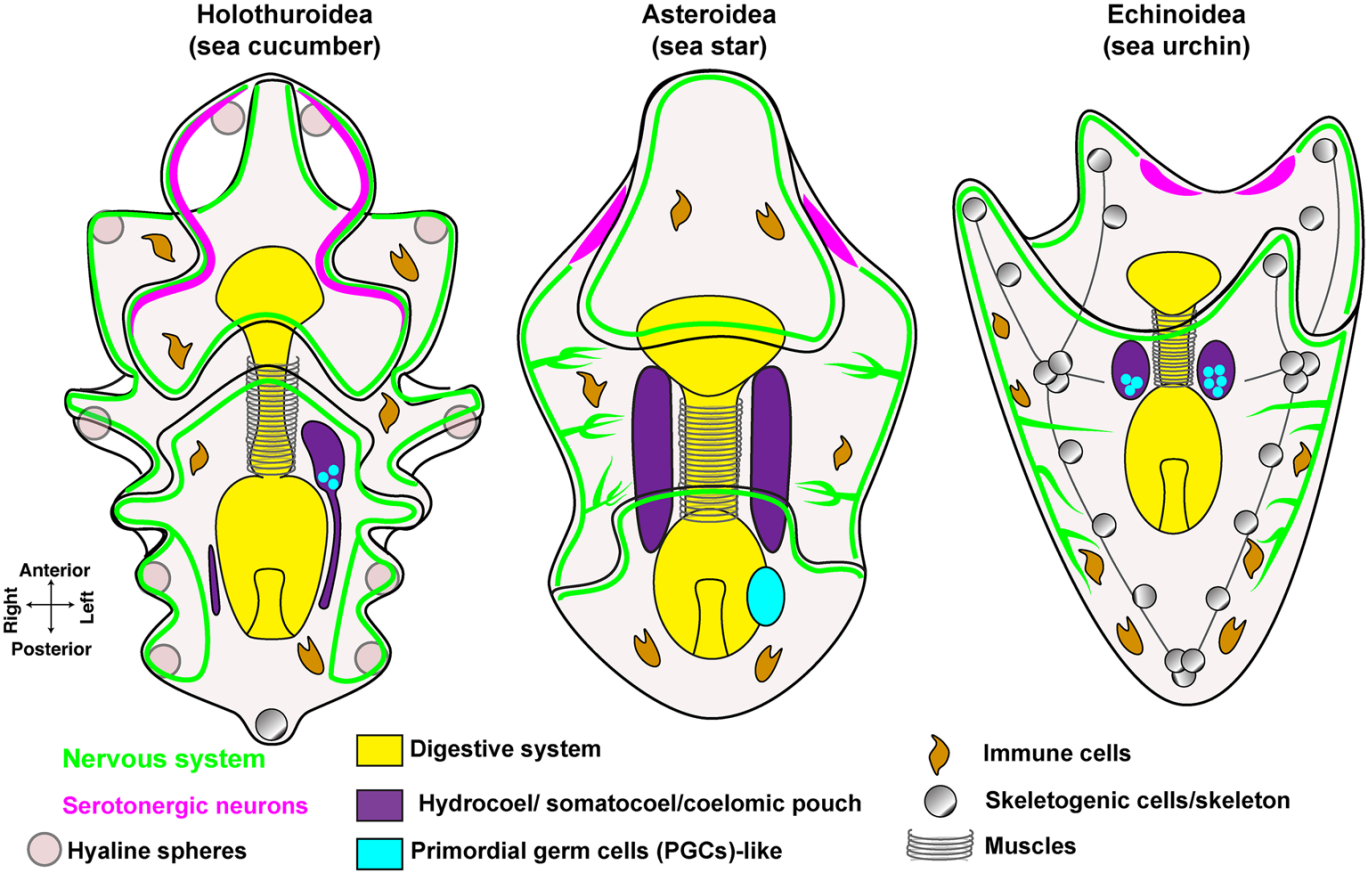
Comparison of the main cell types in the sea cucumber auricularia larva with the sea star bipinnaria and sea urchin pluteus larvae. The cartoons depict the main characterized cell types in echinoderm larvae. Sea cucumber larvae have unique features that distinguish them from other echinoderms, like the presence of hyaline spheres (for lipid storage), one or more short skeletal rods, or ossicles, that do not extend (number varies based on the genus), a single hydrocoel that appears on the left side of the stomach and an extensive neuronal network that do not show long axonal projections towards the internal structures

The holothurian nervous system complexity increases as development continues and neurons are fully differentiated at the feeding auricularia stage. The auricularia nervous system consists of serotonergic ganglia located in the apical organ and neurons distributed along the ciliary band and in close proximity to the esophagus, intestine and anus [27, 110, 178]. Although the overall nervous system organization of holothurians is similar to echinoids and asteroids, several differences have been reported (Fig. 6). For instance, ciliary band neurons project axons towards the aboral ectoderm in sea urchin and toward the oral ectoderm in sea star larvae [66], while holothurians lack this neuronal organization. The fact that the nervous system does not extend towards the ectoderm in holuthurians suggests that this might be a derived character that was not present in the ancestral echinoderm larvae, or that it was lost in sea cucumbers. Interestingly, the localization of serotonergic neurons also shows some differences within echinoderms (Fig. 6): in the sea star larvae these cells form scattered clusters placed laterally on the oral hood [33]; in the echinoid plutei serotonergic neurons are organized in bilateral patches in the apical organ [27] and in holothuroids, these neurons are located close to the ciliary band [110]. It would be interesting to understand if these morphological differences also reflect distinct roles for this cell type in the different echinoderm larvae.

A great diversity of neuropeptides is detected while the auricularia feeds and grows, suggesting their involvement in feeding behavior and larval growth [178]. At this stage, neuropeptides with a role in feeding control in other organisms are present, such like orexin, insulin-like and SALFamide neuropeptides [178]. Less is known about the nervous system organization in the doliolaria and pentactula stages. At the auricularia stage, the nervous system undergoes a dramatic morphological transition as the ciliary bands break to transform into the five ciliary rings of doliolaria (cartoon in Fig. 4), a ring structure that is shared for instance with sea lily larvae [6]. This transformation has been described in *Holothuria mexicana* and *Stichopus californicus* [84]. The doliolaria nervous system also contains dopaminergic and GABAergic neurons associated with sensory structures including ciliated cells [112]. These neurotransmitters are involved in the regulation of ciliary beating and swimming behavior in echinoderms and other marine invertebrates [39, 80, 96], therefore an open question is whether the crosstalk of serotonergic, dopaminergic and GABAergic systems regulates these behaviors in holothurians. Moreover, the presence of GABA and dopamine in sensory organs at the pentactula stage suggests that the settlement and subsequent metamorphosis of the larvae is under the control of dopaminergic and GABAergic systems, another feature potentially shared between marine larvae [39]. Finally, while the nervous system of the doliolaria and pentactula larvae continues to develop, the adult nervous system begins to form, and after metamorphosis it adopts the typical echinoderm organization into radial nerves and nerve rings [110].

Altogether, the nervous system of sea cucumber larvae shares similar developmental features with other echinoderms until the auricularia stage (with some differences in the anatomical distribution of neurons). Future studies should address how this system responds to the environmental stimuli faced by a planktotrophic larva in the water column. Furthermore, the dramatic rearrangement of the nervous system at the doliolaria stage raises important questions. What cellular and molecular factors are involved in the rearrangement of the linear ciliary band neurons into rings? What is the evolutionary advantage of such a circular structure? How do lecithotrophic larvae that do not feed and skip some larval stages sense their environment? We predict that comparisons of the nervous system of echinoderm larvae with different life strategies together with data from other early branched deuterostomes will help unraveling the origins of the bilaterian nervous system.

### Mesodermal cell lineage: immune system, hydrocoel and primordial germ cells

Mesodermal precursors in echinoderms give rise to several larval cell types such as muscles, immune cells, skeleton and coeloms (Fig. 6). Orthologs of genes that in the sea star and sea urchin embryos are expressed by immune and blastocoelar cells (i.e., *erg*, *ets1*, *gata4/5/6*, *foxn2/3*, *tbr*, *tgif* and *gata1/2/3*) are expressed in analogous domains of holothurian embryos (Fig. 5). Studies carried out in *A. japonicus* demonstrated that immune cells are actively involved in immune defense [165], as suggested by the increased expression of three immune-related genes [mannan-binding C-type lectin (MBCL), lysozyme and serine proteinase inhibitor (SPI)] upon exposure to bacterial antigens. Interestingly, several of these immune genes were detected in early developmental stages such as unfertilized egg and pre-hatched blastula, suggesting that either the immune system components are maternally provided to ensure their availability when needed, or that eggs and early zygotes use these molecules in other processes.

The hydrocoel (also named axohydrocoel, anterior coelom or hydro-vascular organ) is an organ unique of echinoderm larvae and it supports the establishment of the pentaradial symmetry during metamorphosis. It also gives rise to the adult water vascular system after metamorphosis, a system that enables circulation and locomotion [17, 74]. This organ is particularly enlarged in the sea star larva, where it originates from mesodermal precursors, forms two tubes on the sides of the gut and eventually elongates into a tubular-shaped organ vital for buoyancy in the water column [17, 118]. Holothurians diverge from other echinoderms since the hydrocoel first arises as a single pouch positioned on the left side of the digestive tract and eventually two somatocoels appear on the left and right sides of the stomach (arrow in Figs. 3i, 4 and [154, 155]). The early morphogenetic changes that lead to this structure in sea cucumber larvae have not been defined yet. Does this single left structure originate from cells of the growing archenteron, or is it made by mesenchyme cells? Which cells give rise to the right somatocoel?

During the shift from a bilateral larva to a pentameric juvenile, the hydrocoel of sea cucumbers forms five lobes (that will give rise to the adult water vascular system) and represents the first pentameric structure before metamorphosis. Lobe formation involves tissue remodeling and is independent from cell proliferation [154, 155]. Using laser ablation, it has been shown that these five lobes give rise to the water vascular system, suggesting it might function as a scaffold for pentameric body formation [154, 155]. What are the gene regulatory modules that lead to the change of symmetry? Is this dramatic shift dependent on some signals coming from the left side of the late larva? These findings, compared with similar studies in the other classes, might shed light on the origins of the molecular mechanisms at the base of pentameral symmetry emergence in echinoderms.

Another critical cell lineage for which the developmental origins in the sea cucumber remain poorly defined are the primordial germ cells (PGCs). Germ line and stem cell-like gene transcripts are enriched in the coelomic pouches of the sea urchin pluteus and in the posterior coelom of the sea star larva [119, 163]. In both animals, these cells enriched in PGCs marker gene transcripts will become part of the somatocoel and therefore will be transmitted to the adult through metamorphosis. The mechanisms of PGC specification in sea cucumbers are still unknown. The transcripts of the conserved germline marker gene *nanos* (encoding an RNA binding protein) are enriched in the hydrocoel of the sea cucumber *A. japonicus* [54]*.* On the other hand, the expression of the RNA helicase Vasa, another marker of stem and germ cells, is not restricted to the hydrocoel and it extends to most larval tissues [54, 170]. In the adults, *vasa* is expressed in both oogonia and spermatogonia, consistent with its conserved role in germ cell differentiation [170]. Functional studies with RNA interference showed that a conserved stem cell and germ-line marker, the P-element induced wimpy testis (*piwi*) gene, is involved in gametogenesis, as its downregulation affects the expression of sex-related genes [148].

Since the expression of *vasa* and *nanos* alone does not clarify which are the precursors of PGCs and their localization, the expression of additional germ-line related genes must be explored in sea cucumbers. Moreover, the factors that are responsible to set aside the PGCs are unknown. PGCs in sea urchins are specified early in development, while in the sea stars PGCs are induced later by cell signaling interactions. Understanding how PGCs develop in sea cucumbers will be critical to define the ancestral mechanism of PGCs specification in echinoderms.

### The evolution of a mineralized skeleton in echinoderm larvae

A striking difference among living echinoderm larvae is the unique presence of a mineralized skeleton in two specific lineages, the echinoids (sea urchins), and the ophiuroids (brittle stars), while sea star and sea cucumber larvae completely and partially lack a skeleton, respectively (Fig. 6). This diversity provides the opportunity to address how novelties arise during animal evolution and undergo different evolutionary paths in animals belonging to the same phylum.

Most of the available information on embryonic and larval skeletogenesis come from echinoids where the skeleton GRN has been reconstructed in detail [49, 113]. In sea urchins, skeletogenic cells migrate from the vegetal plate to the blastocoel during gastrulation. Through a combination of intrinsic mechanisms and signals originating from the surrounding ectoderm, skeletogenic cells fuse into a syncytium and form a ring-like structure around the gut [68, 103]. This scaffold further develops into skeletal rods, beneath the larval ciliated arms, providing support and protection to the developing larvae. Several genes that are essential for skeletogenesis have been identified, the most studied being the master regulator aristaless homeobox *alx1* [50]. Interestingly, the larval skeletogenic GRN of echinoids and ophiuroids, while similar at many levels, underwent specific rewiring events. Furthermore, based on the similarities in their GRNs, it has been proposed that echinoderm skeletogenesis and vertebrate vascularization are controlled by an ancestral tubulogenesis program established in their common ancestor [107].

Since sea cucumber auricularia larvae possess skeletal primordia that do not grow into long rods [101, 134], holothurians represent a unique case to study the evolution of skeletogenesis. Similar to the sea urchin, sea cucumber skeletogenic cells derive from the mesoderm and are among the first cells to ingress during gastrulation, before migrating to the dorsal sides of the embryo to form the cell clusters producing the skeleton [74]. The sea cucumber ortholog of *alx1* has a conserved function in skeletogenesis [32, 101] (Fig. 5), as in sea urchins. The specification of sea cucumber mesodermal cells can be thus considered as an intermediate state between the sea urchin and the sea star and its regulatory landscape has been proposed to be that of the echinoderm ancestor [101]. Understanding how the skeletal ossicle forms is the first step to investigate its contribution to larval biology. How did the skeleton evolve and what is its impact on the overall body morphology? The long arms of sea urchin and brittle star larvae are supported by long skeletal rods, while sea cucumbers that lack such long structures do not develop arms. What are the consequences of these differences for larval shape, swimming behavior, orientation in the water column and feeding? What are the genetic and cellular mechanisms that drive skeleton and arms elongation? From a biophysics perspective, it would be interesting to investigate whether the ossicle affects the swimming dynamics of the sea cucumber larvae and to compare these aspects to the swimming behavior of the late larvae of some asteroid species, like *Pisaster ochraceus*, that develop skeleton-less arms [56]. If the posterior ossicles of the auricularia do not have a clear supporting function, what is the primary function of a larval skeleton in echinoderms? In other words, does the skeleton influence the evolution of diverse larval forms? With the help of genomic tools for functional experiments, we propose that further studies should take advantage of the sea cucumber's unique characteristics of having an under-developed skeleton and investigate, together with other echinoderm larvae, whether a supporting skeleton influences larval shapes and behavior.

## Genomic-transcriptomic information and cellular and molecular tools

Although the molecular regulation of sea cucumber development is understudied compared to other echinoderms, the majority of sequencing projects carried out in these animals represent a significant advantage for researchers willing to use these animals for evo-devo studies.

Species identification has been possible thanks to the complete mitochondrial genome sequencing that allows to resolve phylogenetic relationships. So far, 43 complete mitogenomes have been sequenced from 10 holothurian families, collected in several places worldwide (reported in Additional file 1: Table S1). In addition, 24 genomes have been submitted to GeneBank, 10 of which lack a dedicated publication (Additional file 1: Table S2). The first draft genome of a sea cucumber was performed by Jo and colleagues in 2017 [77], using the Illumina HiSeq 2000 platform. This work provided a general overview of the genetic variation in the three major color variants of *A. japonicus* (green, red, and black), identifying million heterozygous single nucleotide polymorphisms in the assembled genome.

Recently, using PacBio HiFi long-reads and Hi-C sequencing approaches, Sun and collaborators reassembled the *A. japonicus* genome with the aim to provide a chromosome-level assembly for this species [147]. Moreover, to investigate the phenotypic divergence and the population genetic structure of Russian and Chinese *A. japonicus*, the genomes of 210 individuals from the two geographic locations have been fully sequenced [61]. Furthermore, phylogenetic and comparative genomic analyses, using Illumina and PacBio platforms in *A. japonicus* [173] and Nanopore MinION in *H. scabra* [92], have led to the identification of marker genes associated with notochord and gill slits, suggesting that molecular traces of these features can be found in echinoderm cell types. BUSCO assessment of *H. glaberrima* genome allowed to fully annotate the genomic *loci* of the melanotransferrin (Mtf) gene family, which has a potential role in the regeneration of sea cucumber intestine [104].

A recent study reported the assembly and annotation of the *Stichopus monotuberculatus* genome, providing a new genomic approach to study the structural diversity of holothurian genes involved in fucosylated chondroitin sulfates (FCS) biosynthetic pathways [180]. The authors found several expanded gene families, including key enzymes associated with FCS biosynthesis, such as fucosyltransferases and sulfotransferases. They also found FCS genes exclusive to *S. monotuberculatus* providing novel perspectives into the evolutionary adaptation of critical genes in holothurian FCS biosynthesis.

Another study reported the first high-quality, chromosome-level genome assembly of *Holothuria leucospilota*, an ecologically significant sea cucumber species with a prototypical Cuvierian organ (CO), that is a defensive organ with bioadhesive properties [35] The *H. leucospilota* genome reveals characteristic long-repeat signatures in CO-specific proteins, analogous to fibrous proteins of disparate organisms, including spider spidroin and silkworm fibroin, offering new insights into the molecular features and evolution of this unique defensive organ. Likewise, integrated genome-wide association study (GWAS) and the analysis of the distribution of characteristic sex-specific SNPs have clarified sex determination mechanisms and identified sex-linked markers, showing that multiple sex-associated *loci* are located on several chromosomes in *A. japonicus* [158].

Several genomic studies have analyzed the ability of sea cucumbers to adapt to extreme environmental conditions, such as deep-sea, coldness and high pressure hadal zones. For instance, the sequencing of the hadal sea cucumber *Paelopatides sp. Yap* genome helped to identify the potential adaptation mechanisms of these animals to the deep-sea habitat. The authors found in this species an expansion and a positive selection for genes such as translation initiation factors, ribosomal proteins, and genes associated with DNA repair, suggesting that increased protein synthesis inhibition coupled with DNA protection are necessary for deep-sea species adaptation [135]. The sequencing of *C. heheva* genome, another deep-sea species, showed an expansion of the aerolysin-like protein family (pore-forming proteins mostly studied in bacteria and able to damage membranes of target cells generating transmembrane pores) and a positive selection of several hypoxia-related genes, suggesting an important contribution of these genes to hypoxic environment adaptation [172]. These findings in the sea cucumber genome represent important steps towards a better understanding of how deep-sea animals live. For instance, which cell types express the genes involved in deep-sea adaptation in species that live at different depths? Are the same mechanisms of adaptation to hypoxic environments used in both the adult and the embryo?

Finally, the raw genome data of *H. tubulosa* have been recently released, representing a valuable resource for future comparative genomic investigations within the holothurian group [83].

Genome sequencing has been also extremely useful to study epigenetic modifications, in particular *A. japonicus* was used to study DNA methylation and acetylation in response to different experimental conditions (Additional file 1: Table S2). For example, high-resolution methylome analyses by whole-genome bisulfite sequencing (WGBS) showed variations in DNA methylation in the intestine during environmental-induced aestivation [168]. Variations of methylation were also found on healthy body wall and on skin ulceration syndrome infected body wall in *A. japonicus* [146]. Finally, ChIP-seq analysis in *A. japonicus* showed that histone lysine acetylation is a central chromatin modification for gene expression regulation during heat stress response [161].

Several transcriptomic projects are available for *A. japonicus* and *H. scabra*, covering the whole embryonic development up to the larval and juvenile stages (Additional file 1: Table S3) and nicely complementing the genomic resources from these species. In particular, transcriptomes are available for blastula, gastrula, auricularia, pentactula and juvenile stages and have revealed stage-specific transcription factors [25, 47, 114, 156]. RNA sequencing across 16 *A. japonicus* developmental time points (from fertilized egg to juvenile stage) revealed genes involved in early metamorphosis and differentially expressed between late auricularia and doliolaria larvae [88, 89]. Additionally, transcriptional analyses explored the molecular mechanisms that underlie the initial differentiation and formation of papillae in *A. japonicus* by comparing the gene transcriptional profiles of pentactulae (the stage before papillae arise) to those of juveniles (after papillae formation) [171]. Other sequencing analysis have highlighted the genetic basis of saponin biosynthesis, aestivation and regeneration processes. In particular, the transcription factors Klf2 and Egr1 were identified as putative key regulators during *A. japonicus* aestivation (a physiological state characterized by prolonged inactivity, feeding cessation, intestine degeneration and metabolic rate depression, in response to high temperatures) and diverse signaling pathways including Wnt, Hippo and FGF were found to be involved in intestine regeneration [88, 89].

Several biological aspects of adult holothurians have been investigated using RNAseq-based approaches. A series of studies performed differential gene expression analysis of *A. japonicus* subjected to environmental changes such as different light stimuli [90], different salinity conditions [173], and copper exposure [87]. Because sea cucumbers show the remarkable ability of quickly replacing injured organs, transcriptomes have been generated on *H. glaberrima, E. fraudatrix*, and *A. japonic*us during gut evisceration and regeneration [44, 115, 123, 124, 136, 144, 145], radial organ complex regeneration [109], and muscle regeneration [111]. Other studies explored gene expression during aestivation, a process that so far has been exclusively observed in sea sponges and sea cucumbers [159, 174–176]. Moreover, transcriptomes exploring the response of the immune system have been generated in different conditions, such as air exposure stress [150], continuous heat stress [162], skin ulceration [167], microplastic [105] and nanoplastic toxicity [177], miRNA regulation in coelomocytes during host–pathogen interaction [179] and lipopolysaccharide treatment [109], knock-down of *ajpacifastin-like* gene [93], and exposure to *Vibrio splendidus* [130]. All these studies found novel and known genes involved in evisceration/regeneration-related processes, wound healing, cell proliferation, differentiation, morphological plasticity, cell survival, stress response, immune challenge, and neoplastic transformation. Among those, cytoskeletal genes, such as *actins*, and developmental genes, such as *wnt*, *orpin*, *metalloproteinase*, and *hox* genes, have shown interesting expression profiles during regeneration; Wnt, TGF-β and endocytosis pathways have been found associated with cell proliferation and differentiation after evisceration; FoxO signaling pathway has shown playing important roles in immunoregulation.

Recently, a single cell RNA sequencing (scRNA-seq) project aimed to investigate nervous system cell type diversity in the adult *A. japonicus* (BioProject PRJNA883642) has been submitted to NCBI by the Ocean University of China. Another single cell transcriptome project in the same species has been recently published to clarify the molecular nature of different color morphologies [160]. The authors revealed the existence of two cell groups responsible for sea cucumber body color: melanocytes and quinocytes, more abundant in purple than in green sea cucumbers. In addition, important genes related to pigmentation were identified, expanding our knowledge on the molecular mechanisms regulating distinct pigment formation in echinoderms.

On top of multi-omics approaches, cellular and molecular tools have been set up by different research groups to make sea cucumbers biology experimentally accessible (summarized with references in Table 2). Among these, in situ hybridization and immunohistochemistry protocols have been successfully used to study transcripts and protein cellular localization and functional studies have been performed using knock-down approaches, such as RNAi on adults and morpholino antisense oligonucleotides (MASOs) microinjected in zygotes. Lastly, laser ablation has been proved to be a useful tool in holothurians to study tissue regeneration.

**Table 2:** Established techniques that have been used for cellular, developmental and regenerative biology studies on embryo and adult sea cucumbers

Techniques		Species	References
Immunohisto-chemistry	Adults	*Holothuria mexicana* *Holothuria glaberrima* *Stichopus badionotus * *Cucumaria frondosa* *Apostichopus japonicus* *Holothuria forskali* *Leptosynapta clarki* *Eupentacta fraudatrix* *Holothuria scabra* *Holothuria arguinensis* *Pearsonothuria graeffei* *Bohadschia subrubra * *Holothuria polii*	[55] [55] [55] [70] [75] [43] [69] [99] [2] [98] [52] [52] [31]
	Embryos & larvae	*Apostichopus japonicus* *Holothuria atra* *Parastichopus californicus *	[110] [22] [27]
In situ hybridization	Adults	*Holothuria glaberrima*	[100]
	Embryos & larvae	*Apostichopus japonicus* *Parastichopus parvimensis*	[137] [101]
Tissue explants	Adults	*Apostichopus japonicus *ovary & respiratory tree *Holothuria glaberrima* intestinal cultures *Holothuria glaberrima* radial nerve cord cultures	[157] [19] [42]
Gene expression perturbation	Adults	*Apostichopus japonicus* RNA interference (RNAi) *Holothuria glaberrima* Dicer-substrate small interfering RNA (DsiRNA)	[148] [3]
	Embryos & larvae	*Parastichopus parvimensis * Morpholino antisense oligonucleotide (MASO) injection	[101]
Laser ablation	Embryos & larvae	*Apostichopus japonicus*	[155]

## Conclusions and future challenges

As members of Bilateria, the group of animals with bilateral body symmetry, echinoderms have the unique feature of switching from the bilateral symmetry of the embryonic and larval phases (the bipinnaria of asteroids, the pluteus of sea urchins and ophiuroids and the auricularia of holothuroids) (Figs. 1 and 6), to a pentaradial symmetry in the adult body plan (Fig. 1). Because of their rich morphological variation, echinoderm larvae are a powerful tool to investigate the origins of animal diversity and to understand the mechanisms of body patterning.

Although their development is still underexplored at the molecular level, sea cucumbers have all the features that make them valuable experimental systems, such as ease of collection from the field, inexpensive rearing of adults in laboratories, optically transparent embryos and larvae, abundance of eggs that develop into synchronous cultures of embryos and larvae, and available genomes and transcriptomes. Furthermore, their larval anatomy resembles the tornaria larva of hemichordates [149], sister group of echinoderms in the ambulacrarian clade, and it is for this reason considered the ancestral larval type of echinoderms. Another remarkable trait of sea cucumbers is that larvae store lipids in special structures called hyaline spheres and they use them as source of energy during metamorphosis. Several questions related to these peculiar structures are to be addressed: are the hyaline spheres related to our adipocytes? How conserved are the pathways for lipid metabolism in an early branched deuterostome compared to vertebrates?

While other echinoderm larvae undergo a dramatic metamorphosis where the major larval body axes are lost, sea cucumber larvae seem to conserve their embryonic body axis during the transition to adults, a matter still under debate. This makes sea cucumbers useful experimental tools to study the evolution of axis formation and potentially to unravel the origins of deuterostome ancestral developmental mode.

Although so far fewer genetic tools have been developed for sea cucumbers compared to sea stars and sea urchins (discussed in this review), investing in the use of sea cucumbers as experimental systems will be advantageous for the scientific community interested in comparative studies. There is much to be explored on several aspects of sea cucumber larval biology and ecology and many are the open questions that could be addressed by studying these systems in comparison with members of the other echinoderm classes. How does the unique nervous system of the doliolaria larva develop and function? To what extent are these larvae capable of perceiving environmental signals such as light and food availability and how do they respond and possibly adapt to such cues? Considering the extraordinary regenerative potential of the sea cucumber adults that is the subject of several molecular studies, what are the regenerative mechanisms employed by sea cucumber larvae? To answer these questions, we need to combine molecular and genomic techniques in a few species that are easily accessible by researchers worldwide.

The information summarized in Fig. 5 and Additional file 1: Table S3 clearly shows that most of the data related to developmental gene expression patterns derive from studies performed on mainly two species (*A. japonicus* and *P. parvimensis*), and most embryonic and larval transcriptomes have been generated in only one species (*A. japonicus*). Expanding the array of species used in evo-devo studies is crucial to uncover the ancestral traits of this group of animals. Besides *A. japonicus*, broadly used in Asia, another good candidate species to establish sea cucumbers as evo-devo systems can be *H. tubulosa,* a planktotrophic species highly abundant in the Mediterranean Sea that has clear larvae and reproducible spawning methods [126].

Finally, a future challenge to establish sea cucumbers as systems for evo-devo is that the cellular and molecular biology tools discussed in this review should be complemented by the setup of standardized methods for spawning and for oocyte maturation (perhaps isolating a universal peptide to mature oocytes like the one used for the sea stars), and the possibility to overcome seasonality by breeding animals in laboratory-controlled conditions.

### Supplementary Information

**Additional file 1: Table S1.** List of available sea cucumber mitogenomes. **Table S2.** List of available sea cucumber genomes and epigenomes and relative BioProject identification number. **Table S3.** List of sea cucumber transcriptomes with relative accession number and developmental stage.

## Data Availability

Not applicable.
