# Supplementary material for: Sea cucumbers: an emerging system in evo-devo
Source: EvoDevo. 2024 Feb 17;15:3. doi: 10.1186/s13227-023-00220-0 (PMC10874539; doi:10.1186/s13227-023-00220-0)
Supplement: Supplementary file 1 — Additional file 1: Table S1. List of available sea cucumber mitogenomes. Table S2. List of available sea cucumber genomes and epigenomes and relative BioProject identification number. Table S3. List of sea cucumber transcriptomes with relative accession number and developmental stage. [file 13227_2023_220_MOESM1_ESM.pdf]

Table S1. List of available sea cucumber mitogenomes.

Family	Species	Accession	Geographical location	Reference / Submitter Institute
Chiridotidae	*Chiridota heheva*	MW357261	Haima, South China Sea	Sun et al. 2021
	Chiridota sp. SS-2021	MW357262	Manus Basin, Papua New Guinea	
Caudinidae	*Acaudina molpdioides*	MK05010	Ningde, Fujian province, China	Wang et al., 2019
Cucumariidae	*Cercodemas anceps*	MW044622	Sanya city, Hainan Province, China	Li et al. 2021
	Colochirus quadrangularis	MT108721	NA	Lingnan Normal University (China)
	Cucumaria miniata	NC005929	NA	Scouras et al. 2004
	Pseudocolochirus violaceus	MT587564	Shenzhen, Guangdong province, China	Wang et al. 2020
	Thyonella gemmata	MZ463652	Coast of Lower Grand Lagoon Bay, Florida	Figueroa et al. 2021
Elpidiidae	*Scotoplanes*	LC416624 and LC416626	Otsuchi, Iwate, Japan	Takano et al. 2019
	Peniagone sp. YYH-2013	KF915304	Mariana Trench	Second Institute of Oceanography (China)
Holothuriidae	*Actinopyga echinites*	MN793975	SanYa, HaiNan province, China	Zhong et al. 2020
	Actinopyga lecanora	MW248463	LingShui, HaiNan province, China	Zhong et al. 2021
	Bohadschia argus	OL741685	Qionghai, Hainan Province, China	Ma et al. 2022
	Holothuria edulis	MT084774	NA	Lingnan Normal University (China)
	Holothuria forskali	FN562582	Ferrol, Spain	Perseke et al. 2010
	Holothuria fuscogilva	MZ305460	NA	Institute of Marine Drugs, Guangxi University of Chinese Medicine (China)
	Holothuria glaberrima	NA	Puerto Rico, USA	Medina-Feliciano et al, 2021
	Holothuria hilla	MN163001	Changjiang, Hainan Province, China	Yang et al. 2019
	Holothuria leucospilota	MN276190	Beihai, GuangXi province, China	Zhong et al. 2019
	Holothuria leucospilota	ON584426	Tanjung Sedili Beach, Malaysia	Badrulhisham et al. 2023
	Holothuria (Stauropora) pervicax	MK328500	Jeju Island, Korea	Lee & Shin 2019
	Holothuria (Roweothuria) polii	NC045029	Western coast of Sardinia, Oristano province, Italy	Utzeri et al. 2020
	Holothuria scabra	KP257577	NA	Xia et al. 2016
	Holothuria spinifera	MN816440	NA	Dalian Ocean University (China)
Phyllophoridae	*Neocucumis proteus*	MZ305458	NA	Institute of Marine Drugs, Guangxi University of Chinese Medicine (China)
	Phyllophorella liuwutiensis	MN198190	NA	Fisheries Research Institute of Fujian Province (China)
	Phyllophorus liuwutiensis	MN198190	Xiamen City, Fujian Province, China	Yang et al. 2020
	Phyrella fragilis	MZ305459	SanYa, HaiNan province, China	Zhong et al. 2021
Psychropotidae	*Benthodytes marianensis*	MH208310	Mariana Trench	Mu et al. 2018
Stichopodidae	*Apostichopus japonicus*	NC012616	Oita, Japan and Qingdao, China	Sun et al. 2010
	Isostichopus badionotus	MZ188901	St. Andrews State Park, Florida	Drake et al. 2021
	Parastichopus californicus	KP398509	Vancouver Island, Canada	Zhang et al. 2016
	Parastichopus nigripunctatus	AB525762	NA	National Research Institute of Fisheries and Environment of Inland Sea (Japan)
	Apostichopus parvimensis	KU168761	NA	BGI Education Center (China)
	Stichopus chloronotus	MW218897	Sanya City, Hainan Province, China	Chen et al. 2021
	Stichopus horrens	HQ000092	Xisha islands, Hainan Province, China	Fan et al. 2011
	Stichopus monotuberculatus	MN276189	Beihai, GuangXi province, China	Zhong et al. 2019
	Stichopus naso	MZ469138	Qionghai, Hainan Province, China	Li et al. 2022

	Stichopus ocellatus	NC062943	Dong Fang, Hainan province, China	Zhong et al. 2022
	Stichopus sp.	HM853683	Weizhou island, Guangxi province, China	Fan et al. 2012
	Thelenota ananas	MW548268	NA	Hainan Provincial Key Laboratory of Tropical Maricultural Technologies, Hainan Academy of Ocean and Fisheries Sciences (China)
Synallactidae	*Synallactes*	MT559281	Western Pacific Ocean	Liao et al. 2020
Synaptidae	*Euapta godeffroyi*	LC704718	Nyaung Oo Phee Island, Myanmar	Ogawa et al. 2022

Table S2. List of available sea cucumber genomes and epigenomes and relative BioProject identification number.

Family	Species	BioProject	Reference / Submitter Institute
**Genomes**			
Chiridotidae	*Chiridota heheva*	PRJNA752986	Zhang et al. 2022
Holothuriidae	*Holothuria glaberrima*	PRJNA497079	Medina-Feliciano et al. 2021
	Holothuria scabra	PRJNA542245	Luo et al. 2022
	Actinopyga echinites	PRJNA525093	Iridian Genomes (USA)
	Holothuria glaberrima	PRJNA497079	Iridian Genomes (USA)
	Holothuria leucospilota	PRJNA747844	Chen et al. 2023
	Holothuria tubulosa Gmelin, 1791	PRJNA917268	Kyritsi et al. 2023
Pelagothuriidae	*Enypniastes eximia*	PRJNA609335	Iridian Genomes (USA)
Phyllophoridae	*Pentamera pediparva*	PRJNA609338	Iridian Genomes (USA)
Stichopodidae	*Apostichopus japonicus*	PRJNA335936	Jo et al. 2017
		PRJNA413998	Li et al. 2018
		PRJNA354676	Zhang et al. 2017
		PRJNA812362	Wang et al. 2022
		PRJNA865892	Guo et al. 2023
		SRR22523578	Sun et al. 2023
	Australostichopus mollis	PRJEB10682	Long et al. 2016
	Apostichopus californicus	PRJNA720913	Walla Walla University (USA)
	Apostichopus leukothele	PRJNA525091	Iridian Genomes (USA)
	Apostichopus parvimensis	PRJNA182998	Sea Urchin Genome Sequencing and Analysis Consortium
	Stichopus chloronotus	PRJNA511931	Institute of Oceanology, Chinese Academy of Sciences
	Stichopus horrens	PRJNA551342	Universiti Kebangsaan (Malaysia)
	Stichopus monotuberculatus	PRJNA938157	Zhong et al. 2023
Synallactidae	*Paelopatides sp. YAP*	GSA: CRA003479	Shao et al. 2022
	Paelopatides confundens	PRJNA596744	Iridian Genomes (USA)
**Epigenomes**			
Stichopodidae	*Apostichopus japonicus*	PRJNA643989 - Methylome	Yang et al. 2020
		n.a. - Methylome	Sun et al. 2020
		Data on request - Methylome	Han et al. 2021
		n.a. - ChiPseq	Xu et al. 2022

Table S3. List of sea cucumber transcriptomes with relative accession number and developmental stage.

Family / Species	BioProject / Accession	Developmental stage	Reference / Submitter Institute
Stichopodidae / *Apostichopus japonicus*	PRJNA412138	blastula	Boyko et al. 2019
		gastrula	
		auricularia larva	
		pentactula larva	
	PRJNA553613	zygote	Li et al. 2018
		4 cells	
		morula	
		blastula	
		early/late gastrula	
		early/mid/late auricularia larva	
		metamorph 1/2/3/4	
		doliolaria larva	
		pentaculata larva	
		juvenile	
	SRP135833	pentaculata larva	Zhan et al. 2019
		juvenile	
	PRJNA407836	45 dpf	Wang et al. 2018
	PRJNA76487	embryo (4 and 23 hpf)	Du et al. 2012
		larva (30 hpf; 6,8,10 dpf)	
		juvenile (16 and 22 dpf)	
		juvenile (32 and 37 dpf)	
Holothuriidae / *Holothuria leucospilota*	PRJDB3196	blastula; auricularia larva (2 dpf)	University of Tsukba
		auricularia larva (3 dpf)	
Holothuriidae / *Holothuria scabra*	PRJNA433757	juvenile	Ordoñez et al., 2021

References

Badrulhisham, N. S., Solehin, S. N., Han, M. G., Jahari, P. N. S, Mohd Salleh, F., Mohamed Rehan, A., Kamarudin, K. R. (2023). The mitogenome data of *Holothuria (Mertensiothuria) leucospilota* (Brandt,1835) from Malaysia. *Data Brief*. 47:108968. https://doi.org/10.1016/j.dib.2023.108968

Boyko, A. V., Girich, A. S., Eliseikina, M. G., Maslennikov, S. I., & Dolmatov, I. Y. (2019). Reference assembly and gene expression analysis of *Apostichopus japonicus* larval development. *Scientific Reports*, 9(1), 1131. https://doi.org/10.1038/s41598-018-37755-5

Chen, T., Ren, C., Wong, N. K., Yan, A., Sun, C., Fan, D., Luo, P., Jiang, X., Zhang, L., Ruan, Y., Li, J., Wu, X., Huo, D., Huang, J., Li, X., Wu, F., E, Z., Cheng, C., Zhang, X., Wang, Y., & Hu, C. (2023). The Holothuria leucospilota genome elucidates sacrificial organ expulsion and bioadhesive trap enriched with amyloid-patterned proteins. *Proc Natl Acad Sci U S A*, 120(16), e2213512120. https://doi.org/10.1073/pnas.2213512120

Chen, X., Sun, Y., Zhao, H., Hu, J., Chen, B., Li, H., Huang, W. (2021). Complete mitochondrial genome of a tropical sea cucumber, *Stichopus chloronotus*. *Mitochondrial DNA B Resour*. 6(10):2788-2790. https://doi.org/10.1080/23802359.2021.1967218

Drake, V. I., Kim, E., Nigro, H. G., Bogantes, V. E., Janosik, A. M. (2021). The complete mitochondrial genome of the chocolate chip sea cucumber *Isostichopus badionotus* (Echinodermata: Holothuroidea). *Mitochondrial DNA B Resour*. 6(7):1947-1948. https://doi.org/10.1080/23802359.2021.1937365

Du, H., Bao, Z., Hou, R., Wang, S., Su, H., Yan, J., Tian, M., Li, Y., Wei, W., Lu, W., Hu, X., Wang, S., & Hu, J. (2012). Transcriptome sequencing and characterization for the sea cucumber *Apostichopus japonicus* (Selenka, 1867). *PloS one*, 7(3), e33311-e33311. https://doi.org/10.1371/journal.pone.0033311

Fan, S., Hu, C., Wen, J., Zhang, L. (2011). Characterization of mitochondrial genome of sea cucumber *Stichopus horrens*: a novel gene arrangement in Holothuroidea. *Sci China Life Sci*. 54(5):434-41. https://doi.org/10.1007/s11427-011-4168-8

Fan, S., Hu, C., Zhang, L., Sun, H., Wen, J., Luo, P. (2012). Complete mitochondrial genome of the sea cucumber *Stichopus sp*. and its application in the identification of this species. *Aquac. Res*. 43: 1306-1316. https://doi.org/10.1111/j.1365-2109.2011.02934.x

Figueroa, A. C., McHugh, W. J., Miller, S. M., Fellgren, A. K., Bogantes, V. E., Janosik, A. M. (2021). Characterization of the complete mitochondrial genome of *Thyonella gemmata* (Echinodermata: Cucumariidae). *Mitochondrial DNA B Resour*. 6(10):2997-2998. https://doi.org/10.1080/23802359.2021.1975512

Guo, C., Zhang, X., Li, Y., Xie, J., Gao, P., Hao, P., Han, L., Zhang, J., Wang, W., Liu, P., Ding, J., & Chang, Y. (2023). Whole-genome resequencing reveals genetic differences and the genetic basis of parapodium number in Russian and Chinese *Apostichopus japonicus*. *BMC Genomics*, 24(1), 25. https://doi.org/10.1186/s12864-023-09113-x

Han, L., Sun, Y., Cao, Y., Gao, P., Quan, Z., Chang, Y., Ding, J. (2021). Analysis of the gene transcription patterns and DNA methylation characteristics of triploid sea cucumbers (*Apostichopus japonicus*). *Sci Rep*. 11(1):7564. https://doi.org/10.1038/s41598-021-87278-9

Jo, J., Oh, J., Lee, H. G., Hong, H. H., Lee, S. G., Cheon, S., Kern, E. M. A., Jin, S., Cho, S. J., Park, J. K., Park, C. (2017). Draft genome of the sea cucumber *Apostichopus*

japonicus and genetic polymorphism among color variants. *Gigascience*. https://doi.org/10.1093/gigascience/giw006

Kyritsi, M., Tsiolas, G., Michailidou, S., Koukaras, K., & Argiriou, A. (2023). Genomic and 16S metabarcoding data of Holothuria tubulosa Gmelin, 1791. *Data Brief*, 48, 109171. https://doi.org/10.1016/j.dib.2023.109171

Lee, T., Shin, S. (2019). Complete mitochondrial genome of sea cucumber, *Holothuria (Stauropora) pervicax* (Holothuroidea, Holothuriida, Holothuriidae), from Jeju Island, Korea. *Mitochondrial DNA Part B*. 4:1,1047-1048. https://doi.org/10.1080/23802359.2019.1584058

Li, H., Liu, J., Wang, S., Huang, W. (2021). The complete mitochondrial genome of Pink warty sea cucumber (*Cercodemas anceps* Selenka, 1867). *Mitochondrial DNA B Resour*. 6(3):959-961. https://doi.org/10.1080/23802359.2021.1891979

Li, Y., Kikuchi, M., Li, X., Gao, Q., Xiong, Z., Ren, Y., Zhao, R., Mao, B., Kondo, M., Irie, N., & Wang, W. (2018). Weighted gene co-expression network analysis reveals potential genes involved in early metamorphosis process in sea cucumber *Apostichopus japonicus*. *Biochem Biophys Res Commun*, 495(1), 1395-1402. https://doi.org/10.1016/j.bbrc.2017.11.154

Li, Y., Wang, R., Xun, X., Wang, J., Bao, L., Thimmappa, R., Ding, J., Jiang, J., Zhang, L., Li, T., Lv, J., Mu, C., Hu, X., Zhang, L., Liu, J., Li, Y., Yao, L., Jiao, W., Wang, Y., Lian, S., Zhao, Z., Zhan, Y., Huang, X., Liao, H., Wang, J., Sun, H., Mi, X., Xia, Y., Xing, Q., Lu, W., Osbourn, A., Zhou, Z., Chang, Y., Bao, Z., & Wang, S. (2018). Sea cucumber genome provides insights into saponin biosynthesis and aestivation regulation. *Cell discovery*, 4, 29. Retrieved 2018, fromhttp://europepmc.org/abstract/MED/29951224. https://doi.org/10.1038/s41421-018-0030-5

Li, Z., Ma, B., Li, X., Lv, Y., Jiang, X., Ren, C., Hu, C., Luo, P. (2022). The Complete Mitochondrial Genome of *Stichopus naso* (Aspidochirotida: Stichopodidae: *Stichopus*) and Its Phylogenetic Position. *Genes (Basel)*. 13(5):825. https://doi.org/10.3390/genes13050825

Liao, M., Li, B., Xiao, N., Kong, M., Wang, Y., Wang, J., Rong, X., Zhang, Z., Yu, Y. (2020) Complete sequence of mitochondrial DNA of a deep-sea holothurian species of the genus *Synallactes* (Synallactida: Synallactidae). *Mitochondrial DNA B Resour*. 5(3):2699-2700. https://doi.org/10.1080/23802359.2020.1787266

Long, K. A., Nossa, C. W., Sewell, M. A., Putnam, N. H., Ryan, J. F. (2016). Low coverage sequencing of three echinoderm genomes: the brittle star *Ophionereis fasciata*, the sea star *Patiriella regularis*, and the sea cucumber *Australostichopus mollis*. *Gigascience*. 5:20. https://doi.org/10.1186/s13742-016-0125-6

Luo, H., Huang, G., Li, J., Yang, Q., Zhu, J., Zhang, B., Feng, P., Zhang, Y., & Yang, X. (2022). De novo genome assembly and annotation of Holothuria scabra (Jaeger, 1833) from nanopore sequencing reads. *Genes Genomics*, 44(12), 1487-1498. https://doi.org/10.1007/s13258-022-01322-0

Ma, B., Li, Z., Lv, Y., E, Z., Fang, J., Ren, C., Luo, P., Hu, C. (2022). Analysis of Complete Mitochondrial Genome of *Bohadschia argus* (Jaeger, 1833) (Aspidochirotida, Holothuriidae). *Animals (Basel)*. 12(11):1437. https://doi.org/10.3390/ani12111437

Medina-Feliciano, J. G., Pirro, S., García-Arrarás, J. E., Mashanov, V., & Ryan, J. F. (2021). Draft Genome of the Sea Cucumber Holothuria glaberrima, a Model for the Study of Regeneration [Brief Research Report]. *Frontiers in Marine Science*, 8. https://doi.org/10.3389/fmars.2021.603410

Mu, W., Liu, J., Zhang, H. (2018). Complete mitochondrial genome of *Benthodytes marianensis* (Holothuroidea: Elasipodida: Psychropotidae): Insight into deep sea

adaptation in the sea cucumber. *PLoS One*. 13(11):e0208051. https://doi.org/10.1371/journal.pone.0208051

Ogawa, A., Hiruta, S. F., Aung, M. M., Fujita, T. (2022). Complete mitochondrial genome of a sea cucumber, *Euapta godeffroyi* (Echinodermata, Holothuroidea, Apodida, Synaptidae). *Mitochondrial DNA B Resour*. 7(8):1457-1459. https://doi.org/10.1080/23802359.2022.2107462

Ordoñez, J. F. F., Galindez, G., Gulay, K. T., & Ravago-Gotanco, R. (2021). Transcriptome analysis of growth variation in early juvenile stage sandfish *Holothuria scabra*. *Comp Biochem Physiol Part D Genomics Proteomics*, 40, 100904. https://doi.org/10.1016/j.cbd.2021.100904

Perseke, M., Bernhard, D., Fritzsch, G., Brümmer, F., Stadler, P. F., Schlegel, M. (2010). Mitochondrial genome evolution in Ophiuroidea, Echinoidea, and Holothuroidea: insights in phylogenetic relationships of Echinodermata. *Mol Phylogenet Evol*. 56(1):201-11. https://doi.org/10.1016/j.ympev.2010.01.035

Scouras, A., Beckenbach, K., Arndt, A., Smith, M. J. (2004). Complete mitochondrial genome DNA sequence for two ophiuroids and a holothuroid: the utility of protein gene sequence and gene maps in the analyses of deep deuterostome phylogeny. *Mol Phylogenet Evol*. 31(1):50-65. https://doi.org/10.1016/j.ympev.2003.07.005

Shao, G., He, T., Mu, Y., Mu, P., Ao, J., Lin, X., Ruan, L., Wang, Y., Gao, Y., Liu, D., Zhang, L., & Chen, X. (2022). The genome of a hadal sea cucumber reveals novel adaptive strategies to deep-sea environments. iScience, 25(12). https://doi.org/10.1016/j.isci.2022.105545

Sun, H., Zhou, Z., Dong, Y., Yang, A., & Jiang, J. (2020). Insights into the DNA methylation of sea cucumber *Apostichopus japonicus* in response to skin ulceration syndrome infection. *Fish Shellfish Immunol*, 104, 155-164. https://doi.org/10.1016/j.fsi.2020.05.005

Sun, L., Jiang, C., Su, F., Cui, W., & Yang, H. (2023). Chromosome-level genome assembly of the sea cucumber *Apostichopus japonicus*. *Scientific Data*, 10(1), 454. https://doi.org/10.1038/s41597-023-02368-9

Sun, S., Sha, Z., Xiao, N. (2021). The first two complete mitogenomes of the order Apodida from deep-sea chemoautotrophic environments: New insights into the gene rearrangement, origin and evolution of the deep-sea sea cucumbers. *Comp Biochem Physiol Part D Genomics Proteomics*. 39:100839. https://doi.org/10.1016/j.cbd.2021.100839

Sun, X. J., Li, Q., Kong, L. F. (2010). Comparative mitochondrial genomics within sea cucumber (*Apostichopus japonicus*): Provide new insights into relationships among color variants. *Aquaculture*. 309:280–285. https://doi.org/10.1016/j.aquaculture.2010.08.001

Takano, T., Ijichi, M., Itoh H., Fukuda, H., Yoshizawa, S. (2019). Complete mitochondrial genome sequences of a deep-sea holothurian species of the genus *Scotoplanes* (Elasipodida: Elpidiidae). *Mitochondrial DNA Part B*. 4:1,112-113. https://doi.org/10.1080/23802359.2018.1536462

Utzeri, V. J., Ribani, A., Bovo, S., Taurisano, V., Calassanzio, M., Baldo, D., Fontanesi, L. (2020). Microscopic ossicle analyses and the complete mitochondrial genome sequence of *Holothuria (Roweothuria) polii* (Echinodermata; Holothuroidea) provide new information to support the phylogenetic positioning of this sea cucumber species. *Mar Genomics*. 51:100735. https://doi.org/10.1016/j.margen.2019.100735

Wang, G., Li, X., Wang, J., Zhang, J., Liu W., Lu, C., Guo, Y., Dong, B., (2019) The complete mitochondrial genome and phylogenetic analysis of *Acaudina molpdioides*. *Mitochondrial DNA Part B*. 4:1, 668-669. https://doi.org/10.1080/23802359.2019.1572476

Wang, Y., Zeng, L., Wen, J., Li, X., Huang, Y., Sun, Y., Zhao, J. (2020). The complete mitochondrial genome of *Pseudocolochirus violaceus* (Cucumariidae, Dendrochirotida). *Mitochondrial DNA B Resour*. 5(3):2805-2806. https://doi.org/10.1080/23802359.2020.1788455

Wang, Y., Yang, Y., Li, Y., & Chen, M. (2022). Identification of sex determination locus in sea cucumber *Apostichopus japonicus* using genome-wide association study. *BMC Genomics*, 23(1), 391. https://doi.org/10.1186/s12864-022-08632-3

Wang, Z., Cui, J., Song, J., Wang, H., Gao, K., Qiu, X., Gou, M., Li, X., Hu, Z., Wang, X., & Chang, Y. (2018). Comparative Transcriptome Analysis Reveals Growth-Related Genes in Juvenile Chinese Sea Cucumber, Russian Sea Cucumber, and Their Hybrids. *Mar Biotechnol (NY)*, 20(2), 193-205. https://doi.org/10.1007/s10126-018-9796-6

Xia, J., Ren, C., Yu, Z., Wu, X., Qian, J., Hu, C. (2016). Complete mitochondrial genome of the sandfish *Holothuria scabra* (Holothuroidea, Holothuriidae). *Mitochondrial DNA A DNA Mapp Seq Anal*. 27(6):4174-4175. https://doi.org/10.3109/19401736.2014.1003899

Xu, D., Fang, H., Liu, J., Chen, Y., Gu, Y., Sun, G., & Xia, B. (2022). ChIP-seq assay revealed histone modification H3K9ac involved in heat shock response of the sea cucumber *Apostichopus japonicus*. *Sci Total Environ*, 820, 153168. https://doi.org/10.1016/j.scitotenv.2022.153168

Yang, F., Zhou, C., Tran, N. T., Sun, Z., Wu, J., Ge, H., Lu, Z., Zhong, C., Zhu, Z., Yang, Q., Lin, Q. (2020). Comparison of the complete mitochondrial genome of *Phyllophorus liuwutiensis* (Echinodermata: Holothuroidea: Phyllophoridae) to that of other sea cucumbers. *FEBS Open Bio*. 10(8):1587-1600. https://doi.org/10.1002/2211-5463.12914

Yang, Q., Lin, Q., Yang, F., Wu, J., Lu, Z., Li, S., Zhou, C. (2019). Characterization of the complete mitochondrial genome of a holothurians species: *Holothuria hilla* (Holothuroidea: Holothuriidae). *Mitochondrial DNA B Resour*. 4(2):2847-2848. https://doi.org/10.1080/23802359.2019.1660267

Yang, Y., Zheng, Y., Sun, L., & Chen, M. (2020). Genome-Wide DNA Methylation Signatures of Sea Cucumber *Apostichopus japonicus* during Environmental Induced Aestivation. *Genes (Basel)*, 11(9). https://doi.org/10.3390/genes11091020

Zhan, Y., Lin, K., Ge, C., Che, J., Li, Y., Cui, D., Pei, Q., Liu, L., Song, J., Zhang, W., & Chang, Y. (2019). Comparative transcriptome analysis identifies genes associated with papilla development in the sea cucumber *Apostichopus japonicus*. *Comp Biochem Physiol Part D Genomics Proteomics*, 29, 255-263. https://doi.org/10.1016/j.cbd.2018.12.009

Zhang, L., He, J., Tan, P., Gong, Z., Qian, S., Miao, Y., Zhang, H. Y., Tu, G., Chen, Q., Zhong, Q., Han, G., He, J., & Wang, M. (2022). The genome of an apodid holothuroid (Chiridota heheva) provides insights into its adaptation to a deep-sea reducing environment. *Commun Biol*, 5(1), 224. https://doi.org/10.1038/s42003-022-03176-4

Zhang, X., Sun, L., Yuan, J., Sun, Y., Gao, Y., Zhang, L., Li, S., Dai, H., Hamel, J. F., Liu, C., Yu, Y., Liu, S., Lin, W., Guo, K., Jin, S., Xu, P., Storey, K. B., Huan, P., Zhang, T., Zhou, Y., Zhang, J., Lin, C., Li, X., Xing, L., Huo, D., Sun, M., Wang, L., Mercier, A., Li, F., Yang, H., & Xiang, J. (2017). The sea cucumber genome provides insights into morphological evolution and visceral regeneration. *PLoS Biol*, 15(10), e2003790. https://doi.org/10.1371/journal.pbio.2003790

Zhang, Z., Bao, X., Dong, Y., Gao, X., Gao, L., Li, S., Liu, W., Hou, H., Shi, J., Pu, H. (2016). Complete mitochondrial genome of *Parastichopus californicus* (Aspidochirotida: Stichopodidae). *Mitochondrial DNA A DNA Mapp Seq Anal*. 27(5):3569-70. https://doi.org/10.3109/19401736.2015.1074222

Zhong, S., Liu, Y., Huang, L., Zhao, Y., Huang, G. (2019). The complete mitochondrial genome of black sea cucumber *Holothuria leucospilota* (Aspidochirotida holothuriidae). *Mitochondrial DNA B Resour*. 4(2):3377-3378. https://doi.org/10.1080/23802359.2019.1673250

Zhong, S., Liu, Y., Zhao, Y., Huang, G. (2019). The complete mitochondrial genome of sea cucumber *Stichopus monotuberculatus* (aspidochirotida: Stichopodidae). *Mitochondrial DNA B Resour*. 4(2):3305-3306. https://doi.org/10.1080/23802359.2019.1673244

Zhong, S., Huang, L., Liu, Y., Huang, G. (2020). The first complete mitochondrial genome of *Actinopyga* from *Actinopyga echinites* (Aspidochirotida: Holothuriidae). *Mitochondrial DNA B Resour*. 5(1):854-855. https://doi.org/10.1080/23802359.2019.1710598

Zhong, S., Qiao, Y., Zhao, L., Huang, G., Liu, Y., Huang, L. (2021). Characterization and phylogenetic analysis of the complete mitochondrial genome of *Actinopyga lecanora* (Jaeger, 1833) (Holothuriida: Holothuriidae). *Mitochondrial DNA B Resour*. 6(10):2801-2802. https://doi.org/10.1080/23802359.2021.1970641

Zhong, S., Zhao, L., Huang, G., Liu, Y., Huang, L. (2021). The first complete mitochondrial genome of *Phyrella fragilis* (Mitsukuri & Ohshima in Ohshima, 1912) (Dendrochirotida: Phyllophoridae). *Mitochondrial DNA B Resour*. 6(10):2979-2980. https://doi.org/10.1080/23802359.2021.1976687

Zhong, S., Zhao, L., Huang, G., Huang, L., Liu, Y. (2022). Characterization and phylogenetic analysis of the complete mitogenome of sea cucumber *Stichopus ocellatus* (Massin, Zulfigar, Hwai & Boss, 2002) (Aspidochirotida: Stichopodidae). *Mitochondrial DNA B Resour*. 7(9):1740-1742. https://doi.org/10.1080/23802359.2022.2124829

Zhong, S., Ma, X., Jiang, Y., Liu, X., Zeng, M., Zhao, L., Huang, L., Huang, G., Zhao, Y., Qiao, Y., & Chen, X. (2023). The draft genome of the tropical sea cucumber *Stichopus monotuberculatus* (Echinodermata, Stichopodidae) reveals critical genes in fucosylated chondroitin sulfates biosynthetic pathway [Data Report]. *Frontiers in genetics*, 14. https://doi.org/10.3389/fgene.2023.1182002
